# Critical Amino Acids within the Human Immunodeficiency Virus Type 1 Envelope Glycoprotein V4 N- and C-Terminals Contribute to Virus Entry

**DOI:** 10.1371/journal.pone.0086083

**Published:** 2014-01-21

**Authors:** Yan Li, Dan Yang, Jia-Ye Wang, Yuan Yao, Wei-Zhe Zhang, Lu-Jing Wang, De-Chun Cheng, Feng-Kun Yang, Feng-Min Zhang, Min Zhuang, Hong Ling

**Affiliations:** 1 Department of Microbiology, Harbin Medical University, Harbin, China; 2 Department of Forensic Medicine, Harbin Medical University, Harbin, China; 3 Academy of Fundamental and Interdisciplinary Science, Harbin Institute of Technology, Harbin, China; 4 Department of Parasitology, Harbin Medical University, Harbin, China; 5 Department of Biochemistry, Harbin Medical University, Harbin, China; 6 Heilongjiang Provincial Key Laboratory for Infection and Immunity, Key Laboratory of Etiology of Heilongjiang Province Education Bureau, Harbin, China; German Primate Center, Germany

## Abstract

The importance of the fourth variable (V4) region of the human immunodeficiency virus 1 (HIV-1) envelope glycoprotein (Env) in virus infection has not been well clarified, though the polymorphism of this region has been found to be associated with disease progression to acquired immunodeficiency syndrome (AIDS). In the present work, we focused on the correlation between HIV-1 gp120 V4 region polymorphism and the function of the region on virus entry, and the possible mechanisms for how the V4 region contributes to virus infectivity. Therefore, we analyzed the differences in V4 sequences along with coreceptor usage preference from CCR5 to CXCR4 and examined the importance of the amino acids within the V4 region for CCR5- and CXCR4-tropic virus entry. In addition, we determined the influence of the V4 amino acids on Env expression and gp160 processing intracellularly, as well as the amount of Env on the pseudovirus surface. The results indicated that V4 tended to have a shorter length, fewer potential N-linked glycosylation sites (PNGS), greater evolutionary distance, and a lower negative net charge when HIV-1 isolates switched from a coreceptor usage preference for CCR5 to CXCR4. The N- and C-terminals of the HIV-1 V4 region are highly conserved and critical to maintain virus entry ability, but only the mutation at position 417 in the context of ADA (a R5-tropic HIV-1 strain) resulted in the ability to utilize CXCR4. In addition, 390L, 391F, 414I, and 416L are critical to maintain gp160 processing and maturation. It is likely that the hydrophobic properties and the electrostatic surface potential of gp120, rather than the conformational structure, greatly contribute to this V4 functionality. The findings provide information to aid in the understanding of the functions of V4 in HIV-1 entry and offer a potential target to aid in the development of entry inhibitors.

## Introduction

The envelope glycoprotein (Env) of human immunodeficiency virus 1 (HIV-1) binds to receptors and triggers conformational changes to initiate viral infection. The entry of HIV-1 into target cells requires fusion between the viral and cellular membranes, which is mediated by the Env [1], [2]. Env is expressed as a heavily glycosylated precursor (gp160) that is cleaved intracellularly into two non-covalently-associated functional subunits: an extracellular subunit (gp120), responsible for CD4 and coreceptor (primarily CCR5 and/or CXCR4) binding, and a transmembrane subunit (gp41) that mediates the fusion between the viral and host cell membranes.

HIV-1 Env is highly variable in five sequence regions (variable regions, V1 to V5) and highly conserved in other regions (conserved regions, C1 to C5). The HIV-1 Env polymorphism reflects the selective pressures of the immune system on viruses [3] and assists in virus escape. In contrast, Env conservation reflects the stability of virus evolution and it is necessary for maintaining virus function. Notably, the gp120 V4 region is highly polymorphic in length, amino acid composition, and glycosylation [4]–[6]. However, the biological significance of the extensive evolution of the V4 region and its association with viral infectivity have not been well established.

Some studies have revealed that the V4 region is associated with disease progression in SIV and HIV infection [7]–[9]. The sequences within the V4-V5 regions in subjects with slow CD4 cell loss were more variable than in subjects with rapid CD4 cell loss [7]. The envelopes isolated from individuals infected with subtype C HIV-1 demonstrated that slow progressors had a significantly longer C3-V4 region compared with progressors [8]. In addition, rapid progressors among SIV-infected macaques exhibited high diversity in the V4 region [9]. Furthermore, neutralization escape has been associated with amino acid substitutions and sequence insertion/deletion in the V4 region in SIV [10], [11] andaqqa HIV infection [12].

During HIV-1 infection, the disease progression is primarily associated with massive damage of target cells caused by the successful entry of viruses and the infection of target cells. It was found that the V4–V5 regions may play an important role in affecting fusion efficiency [13], and the V4 mutations at positions 407D and 386N can modulate resistance to CCR5 antagonists [14]. Moreover, V4 variation correlates with coreceptor preference because a CCR5-tropic virus strain with a V4-V5 region replaced by the V4-V5 region from a CXCR4-tropic virus can utilize CXCR4 [15], and rapid and extensive changes in the amino acid sequences in the V4-V5 regions have also been demonstrated to accompany coreceptor switching from CCR5 to R5X4 [16].

It is still unclear how the highly variable V4 region affects disease progression and whether it is a result of affecting virus entry capacity. The identification of concrete amino acid residues or subregions in the V4 region corresponding to the Env functions to find a potentially novel target for HIV-1 entry inhibitors is worthwhile. In this study, we focused on exploring the correlation between the polymorphism of V4 region and its influence on virus entry, and the possible mechanisms for how the HIV-1 gp120 V4 region contributes to virus infectivity.

## Materials and Methods

### Sequence Collection

All of the available V4 amino acid sequences from individuals infected with HIV-1 clade B with known coreceptor usage were collected from the Los Alamos HIV Sequence Database (http://www.hiv.lanl.gov). Envs with an incomplete open reading frame were discarded. The sequences are generally similar if they were isolated from same individual. Therefore, we selected only one sequence per individual by option provided in the HIV Databases. In total, 190 CCR5-utilizing only, 32 CXCR4-utilizing only, and 30 CCR5CXCR4-utilizing only sequences were collected. GenBank accession numbers of the V4 gene sequences were shown in. According to the coreceptor-usage preference from CCR5 to CXCR4 by HIV-1 isolates along with the progression of viral infection, all of the sequences were divided into two groups: R5 (n = 190) and X4 (R5X4) (n = 62). The X4 (R5X4) group contained sequences that could utilize CXCR4 only or both CXCR4 and CCR5.

### Sequence Alignment and Analysis

All the sequences were aligned using ClustalW (http://www.ebi.ac.uk/Tools/clustalw2/index.html) and manually adjusted for optimal alignment. Subsequently, the frequencies of the amino acids at each position within the sequences were counted, arrayed, and edited in a descending order. The number of potential N-linked glycosylation sites (PNGS) was counted using the NetNGlyc 1.0 Server (http://www.cbs.dtu.dk/services/NetNGlyc/). The net charge was analyzed using GeneRunner version 3.05. The evolutionary distance between each group of sequences and the consensus sequence of subtype B was analyzed using the Pairwise distance calculation method in the MEGA 3.1 program [17].

### Cell Lines

A human embryonic kidney cell line (HEK293T) was purchased from ATCC. HEK293T cells were maintained in DMEM (GibcoBRL, Grand Island, NY, USA) supplemented with 10% fetal calf serum (FCS), 2 mM L-glutamine, 0.1 mM non-essential amino acids (GibcoBRL, Grand Island, NY, USA), 110 mg/l sodium pyruvate (GibcoBRL, Grand Island, NY, USA), 100 µg/ml penicillin, and 100 µg/ml streptomycin (GibcoBRL, Grand Island, NY, USA). Human astroglia cell lines expressing CD4 and the coreceptors CXCR4 (U87.CD4.CXCR4) or CCR5 (U87.CD4.CCR5) were obtained from the AIDS Research and Reference Reagent Program and maintained in DMEM and 10% FCS [15]. We added 300 µg/ml G418 (GibcoBRL, Grand Island, NY, USA) and 1 µg/ml puromycin (Sigma, St. Louis, MO, USA) as the selection reagents for CD4 and the coreceptors.

### Construction of the Env Pseudoviruses and the Infection Assay

Two well-characterized R5-tropic and X4-tropic HIV-1 strains, ADA and HXB2, were used in this study. We constructed a series of gp160 mutants by either deleting a portion or substituting an individual residue of the V4 region and maintaining the two terminal cysteines to retain the loop conformation. To generate luciferase-reporter Env-pseudoviruses, 293T cells were co-transfected with the expressing plasmid pNL4-3-Luc-E-R- (provided by Dr D.R. Littman) and each of the gp160-expressing plasmids [18]. The pseudoviruses were filtered with 0.45 µM membrane filters and quantified using the P24 antigen ELISA assay (ZeptoMetrix Corporation, Buffalo, NY, USA). The coreceptor usage and the infectivity of the pseudoviruses with wild-type (wt) or V4-mutated Env glycoproteins were examined in a single-round infection assay [18]. Pseudoviruses, at 20 ng or 40 ng of P24, were used to infect 2×10^4^ U87.CD4.CCR5 or U87.CD4.CXCR4 cells grown in 48-well plates in triplicate. The luciferase activity (relative light units, RLU) was examined 48 h post-infection using a luminometer (Turner 20/20n, Turner Designs, Mountain View, CA, USA) and the Luciferase Assay System (Promega, Madison, WI, USA). The experiments were performed three to five times. The infectivity was expressed as the percentage of the RLU of ADA-wt or HXB2-wt.

### SDS-PAGE and Western Blotting

To examine the amount of Env glycoproteins expressed in the cells and shed to the supernatant, the cell lysates and supernatants were collected after the cotransfection with the two plasmids as described above. The supernatants, filtered with 0.45 µM membranes, were used to detect gp120 shedding. Moreover, the supernatants were pelleted at 20000 g for 2 hours to harvest the pseudovirus particles for detection of the Env incorporation into surface of pseudovirions. An equal amount of the pelleted pseudoviruses normalized by P24 quantity was loaded into 4–15% gradient precast gels (Bio-Rad, Hercules, CA) and blotted onto PVDF membranes (Millipore, Bedford, MA, USA) [19]. Then, gp120 and gp160 were detected with rabbit anti-gp120 serum (Advanced Biotechnology, USA) at a 1∶1000 dilution, followed by an HRP-conjugated goat anti-rabbit antibody (Zhongshan GoldenBridge Biotechnology, Beijing, China) at a 1∶2000 dilution. The HIV-1 P24 protein expression from each sample in western blot was detected using the murine monoclonal antibody VAK4 to normalize the amount of pseudoviruses [20], followed by an HRP-conjugated goat anti-mouse antibody (Zhongshan GoldenBridge Biotechnology, Beijing, China). The protein bands were visualized using the ECL system (Boster, Wuhan, China), quantified using a FUJIFILM LAS4000 image analyzer and reported as integrated intensities (i.i.) (FuJiFilm, Tokyo, Japan). The expression of gp120 and gp160 in cell lysates was normalized to the amount of GAPDH. The relative amount of gp160 and gp120 was shown as a percentage of wild-type expression. Because gp160 is cleaved into gp120 and gp41, the cleavage efficiency (%) of each Env was expressed by the following formula: (i.i. of gp120)/(i.i. of gp120+ i.i. of gp160). Two to three independent transfections with each of the Env constructs were analyzed.

### Statistical Methods

The differences in the amino acid lengths, PNGS, evolutionary distances, and net charges between the two V4 sequence groups were assessed using the Wilcoxon rank sum test. Correlation between infectivity and cleavage efficiency/amount of gp120 on virions were assessed by using the nonparametric Spearman’s rank correlation test for univariate comparisons. All statistical analyses were performed with SigmaStat 3.0 program and all *P* values were two-sided, and a *P* value of less than 0.05 was considered statistically significant.

## Results

### X4-tropic HIV-1 V4 Regions Tended to have a Shorter Length, Fewer PNGS, Greater Evolutionary Distance, and Less Negative Net Charge Compared with the V4 Regions of R5-tropic Regions

**Figure 1:**
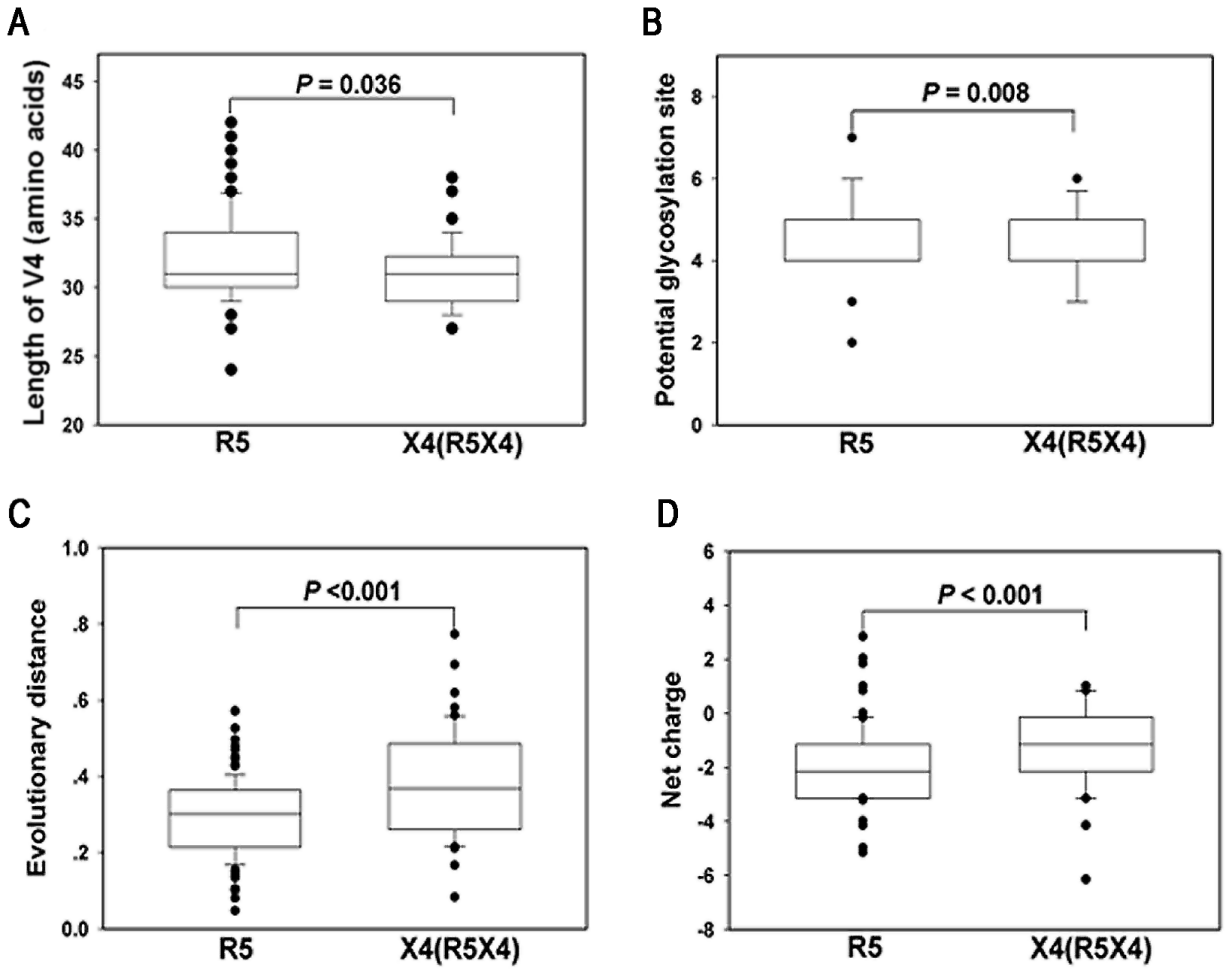
Diversity of the V4 region between R5- and X4 (R5X4)-tropic strains. The differences in amino acid length (A), PNGS (B), evolutionary distance (C), and net charge (D) are depicted in box plot diagrams, in which the box represents the 25th and 75th quartiles, and the line is the median value. The bars extending from each box indicate the 5th and 95th percentiles, and the discs indicate outliers.

We analyzed the amino acid sequence diversity between the V4 regions in R5- and X4 (R5X4)-tropic HIV-1 strains (Figure 1). We found that the V4 regions of the R5-tropic strains (median, 31 residues; range, 24 to 42) were longer than of the X4(R5X4)-tropic strains (median, 31; range, 27 to 38; *P* = 0.036). The number of PNGS in the R5-tropic strains (median, 5; range, 2 to 7) was higher than in the X4 (R5X4)-tropic strains (median, 4; range, 3 to 6; *P* = 0.008). However, the evolutionary distance of the X4 (R5X4)-tropic strains (median, 0.368; range, 0.083 to 0.773) was significantly greater than that of the R5-tropic strains (median, 0.302; range, 0.047 to 0.571; *P*<0.001). The net charge of V4 was more negative in the R5-tropic strains (median, −2.15; range, −5.15 to 2.85) than in the X4 (R5X4)-tropic strains (median, −1.15; range, −6.15 to 1.02; *P* = 0.014).

### HIV-1 V4 Regions are Highly Conserved in N- and C-terminals but Extremely Variable in the Central Subregion

**Figure 2:**
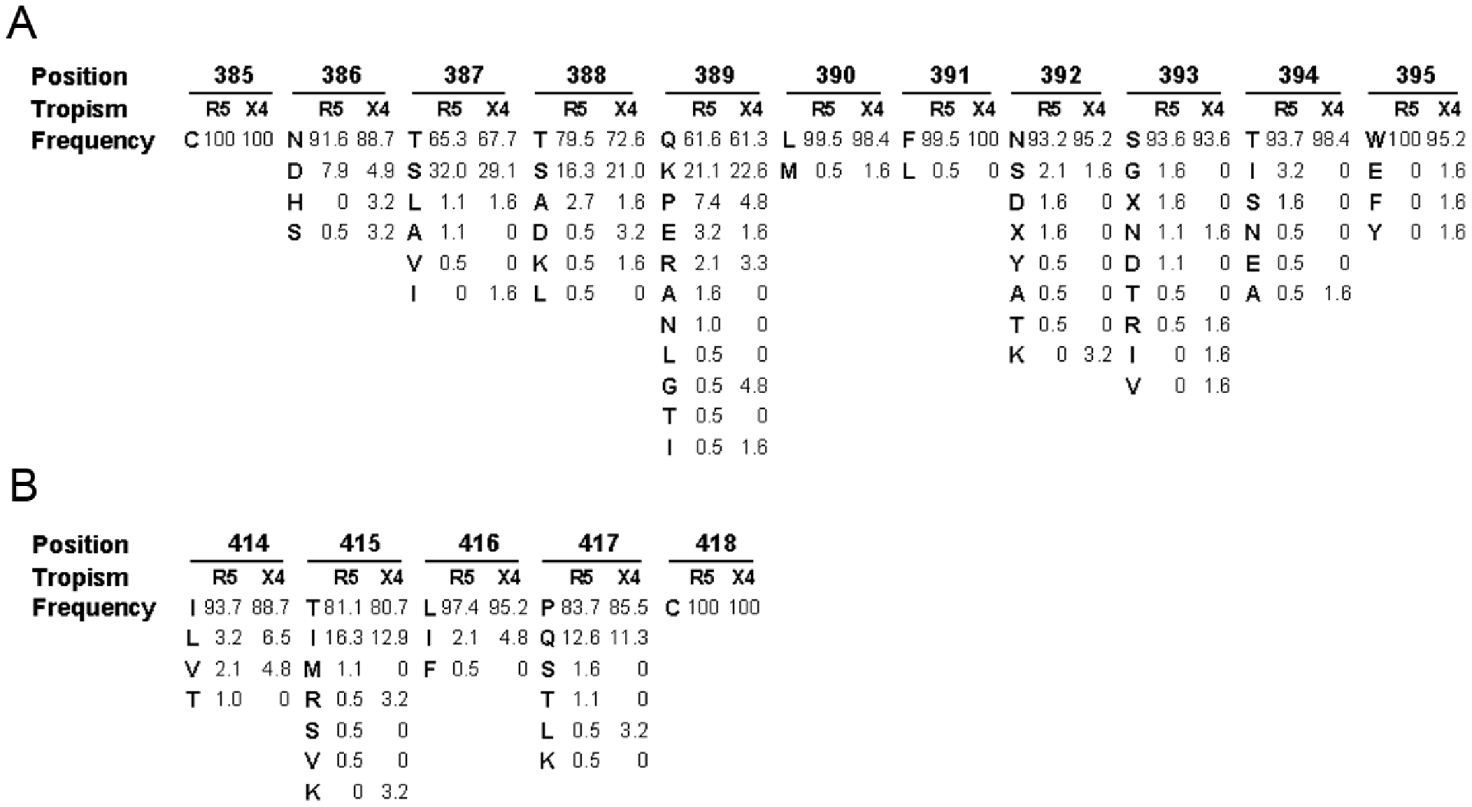
Amino acid frequency at each position of the V4 region in R5- and X4(R5X4)-tropic strains. The site-specific frequency of the amino acid residues at the N-terminal (A) and C-terminal (B) of R5-tropic (R5) and X4(R5X4)–tropic (X4) HIV-1 V4 in descending order is shown. The position aligned is based on HXB2 numbering.

When we tried to align the sequences and analyze the amino acid frequency at each position, we found that the central portions of the V4 regions (amino acids 396–413) among all of the sequences were extremely variable and difficult to align accurately. In contrast, the residues at positions, 386–395 and 414–417, derived from either R5- or X4 (R5X4)-tropic HIV-1 strains, were highly conserved (Figure 2).

Among all of the sequences, the amino acids 390L, 391F, 395W, and 416L were present in more than 95% of the strains. Only leucine and methionine were found at position 390. Leucine was found in 99.5% of the R5-tropic strains and in 98.4% of the X4 (R5X4)-tropic strains. 391F was present in 99.5% of the R5-tropic strains and in 100% of the X4 (R5X4)-tropic strains. Tryptophan was found at position 395, and it was present in 100% of the R5-tropic strains and in 95.2% of the X4 (R5X4)-tropic strains. In addition, 416L was present in 97.4% of the R5-tropic strains and in 95.2% of the X4 (R5X4)-tropic strains. In addition, 392N, 393S, and 394T were also conserved in more than 90% of the strains. However, 387T, 388T, and 389Q were present in approximately 60–80% of the R5- and X4 (R5X4)-tropic strains.

### Shortening of the V4 N- or C-terminal caused the Loss or Decrease of HIV-1 Infectivity

**Figure 3:**
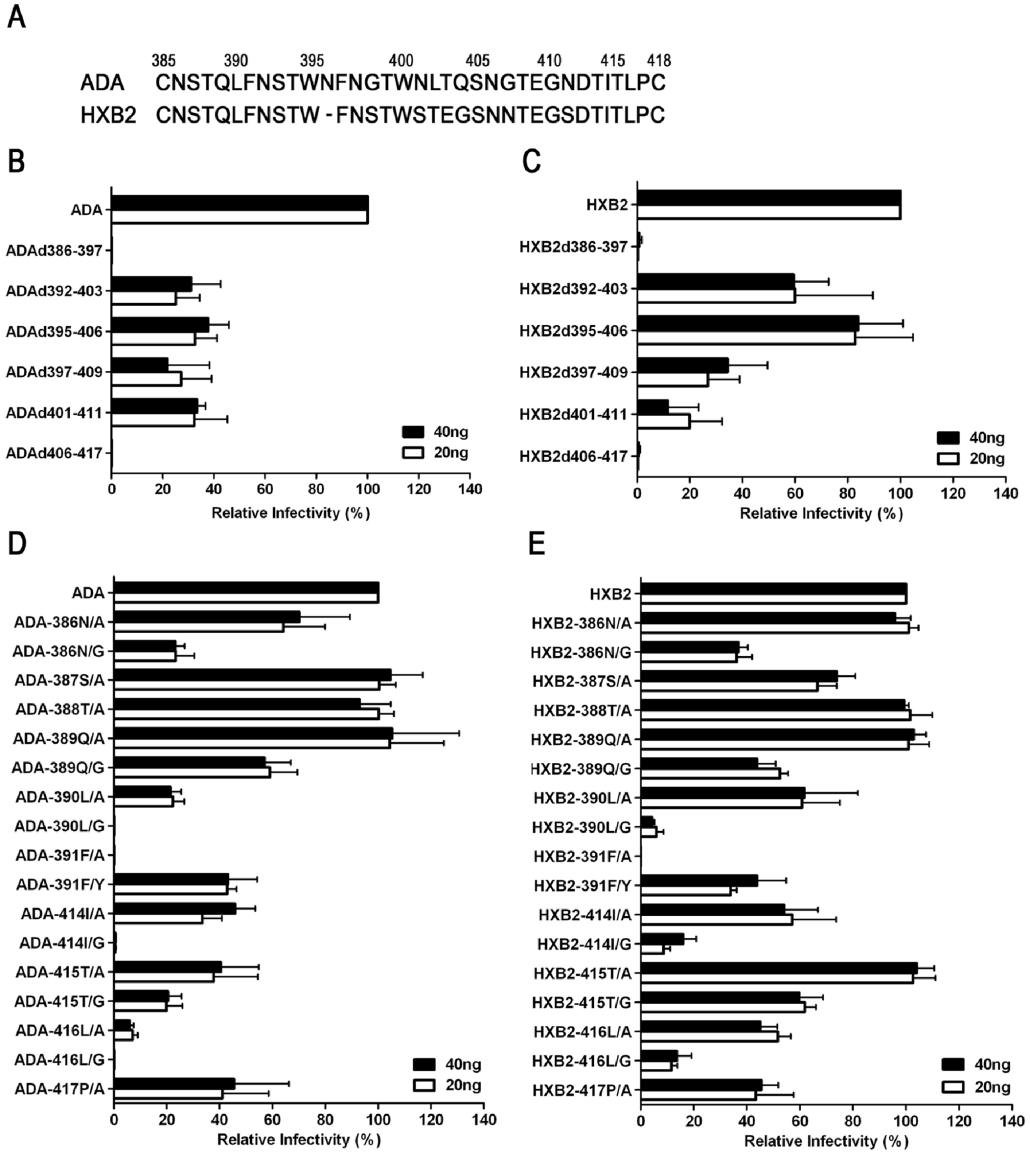
Infectivity of Env pseudoviruses with V4 mutation. The amino acid sequences of V4 derived from ADA and HXB2 are shown in the top panel (A). The number represents the position aligned based on HXB2 numbering. The infectivities of the Env pseudoviruses with partial deletions (B, C) or single amino-acid substitution (D, E) in V4 of ADA (B, D) and HXB2 (C, E) at 20 ng or 40 ng of p24 are expressed as a percentage of ADA-wt or HXB2-wt. The data are shown as the mean±SD of three independent experiments.

The high conservation of the N- and C-terminals of V4 raised the question of whether these subregions are important for Env functions. Therefore, the infectivity of the Env pseudoviruses with a series of partial deletions of V4 was examined (Figure 3A, 3B, and Table 1).

**Table 1:** Summary of Infectivity, Expression, Cleavage Rate and Incorporation of Env into Pseudoviruses (% of HXB2-wt).

Mutant	Infectivity	Total Env	Cleavagerate	Virusgp120
HXB2d386-397	–	+++	–	–
HXB2d392-403	+++	++++	+++	+++
HXB2d395-406	+++	+++	++	+++
HXB2d397-409	+	+++	+++	+++
HXB2d401-411	+	+++	+++	+++
HXB2d406-417	–	+++	–	–
HXB2-386N/A	+++	++++	+++	+++
HXB2-386N/G	++	++++	+++	+++
HXB2-387S/A	+++	+++	+++	+++
HXB2-388T/A	+++	+++	+++	+++
HXB2-389Q/A	++++	++++	++++	+++
HXB2-389Q/G	++	++++	++++	+++
HXB2-390L/A	+++	+++	+++	++
HXB2-390L/G	+	+++	+++	+
HXB2-391F/A	–	+	+	+
HXB2-391F/Y	++	+++	+++	+++
HXB2-414I/A	+++	+++	+++	++
HXB2-414I/G	+	+++	++	+++
HXB2-415T/A	++++	+++	+++	+++
HXB2-415T/G	+++	+++	++++	+++
HXB2-416L/A	++	+++	++	++
HXB2-416L/G	+	+++	+	+
HXB2-417P/A	++	+++	+++	+++

The entry ability and the Env expression of the mutants are shown as the percentage of the wild-type ability/level. –: 0, +: <30%, ++: 30–50%, +++: 50–100%, ++++: >100.

We found that the pseudoviruses with the N- or C-terminal deletions ADAd386-397 and ADAd406-417 completely lost the ability to enter target cells. Similar phenomena were observed with HXB2 mutants. The infectivity of the mutants that contained a series of deletions throughout amino acids 392 to 411 was maintained partially. In addition, ADAd395-406, the mutant with a central portion deleted, displayed infectivity approximately 40% of ADA-wt. However, a mutant, HXB2d395-406, had infectivity similar to HXB2-wt, indicating that the shortening of the V4 central subregion of X4-tropic strains may not damage viral infectivity.

### The Substitution of an Amino Acid at the Conserved Positions in the V4 Region Caused Loss or Decrease of Viral Infectivity

Because the deletion of the N- or C-terminal of V4 caused a complete loss of viral infectivity, we believe that both terminals are critical for virus survival. Therefore, we focused on which individual amino acid (s) in the N- and C-terminals of V4 affect virus entry.

Among all the mutants with a single amino acid substitution in the N- or C-terminal of V4, only 391F/A lost infectivity in the context of both ADA and HXB2 (Figure 3D, 3E, and Table 1). However, the infectivity of the mutant 391F/Y declined to approximately 40% of ADA-wt and HXB2-wt. The infectivity was also impaired when the amino acid 390L, 414I or 416L was substituted by glycine. ADA-390L/G and ADA-416L/G lost entry ability, and the infectivity of ADA-414I/G declined to 1% of ADA-wt. In the context of HXB2, the infectivity of HXB2-390L/G, HXB2-414I/G, and HXB2-416L/G was reduced to less than 20% of HXB2-wt. However, the introduction of alanine as a substitute for 390L, 414I, or 416L did not cause a loss of entry ability. The infectivity of HXB2-390L/A, HXB2-414I/A, and HXB2-416L/A was maintained at 40-70% of HXB2-wt, whereas the infectivity of ADA-390L/A, ADA-414I/A, and ADA-416L/A was maintained at 10-40% of ADA–wt. The elimination of the carbohydrate at position 386 with a 386N/A or 386N/G mutation resulted in lower entry ability, but mutant 388T/A, which also eliminated carbohydrate at position 386, did not damage viral infectivity.

### Coreceptor Usage Switching

To address the question of whether a change in the amino acid residue(s) in the V4 region could cause coreceptor usage switching, we examined the infectivity of the pseudoviruses with mutated HXB2- or ADA- Env to either CCR5- or CXCR4-expressing cells. All the HXB2 pseudoviruses with V4 mutation were not able to infect CCR5-expressing cells. Moreover, among the mutants of the ADA Env, only the mutant ADA-417P/A showed a weak ability to enter CXCR4-expressing cells (the RLU were about 90 times higher than blanks), and still retained a partial ability to infect CCR5-expressing cells (approximately 50% of ADA-wt).

### Cleavage Efficiency of the Env Mutants and Incorporation into Pseudoviruses

**Figure 4:**
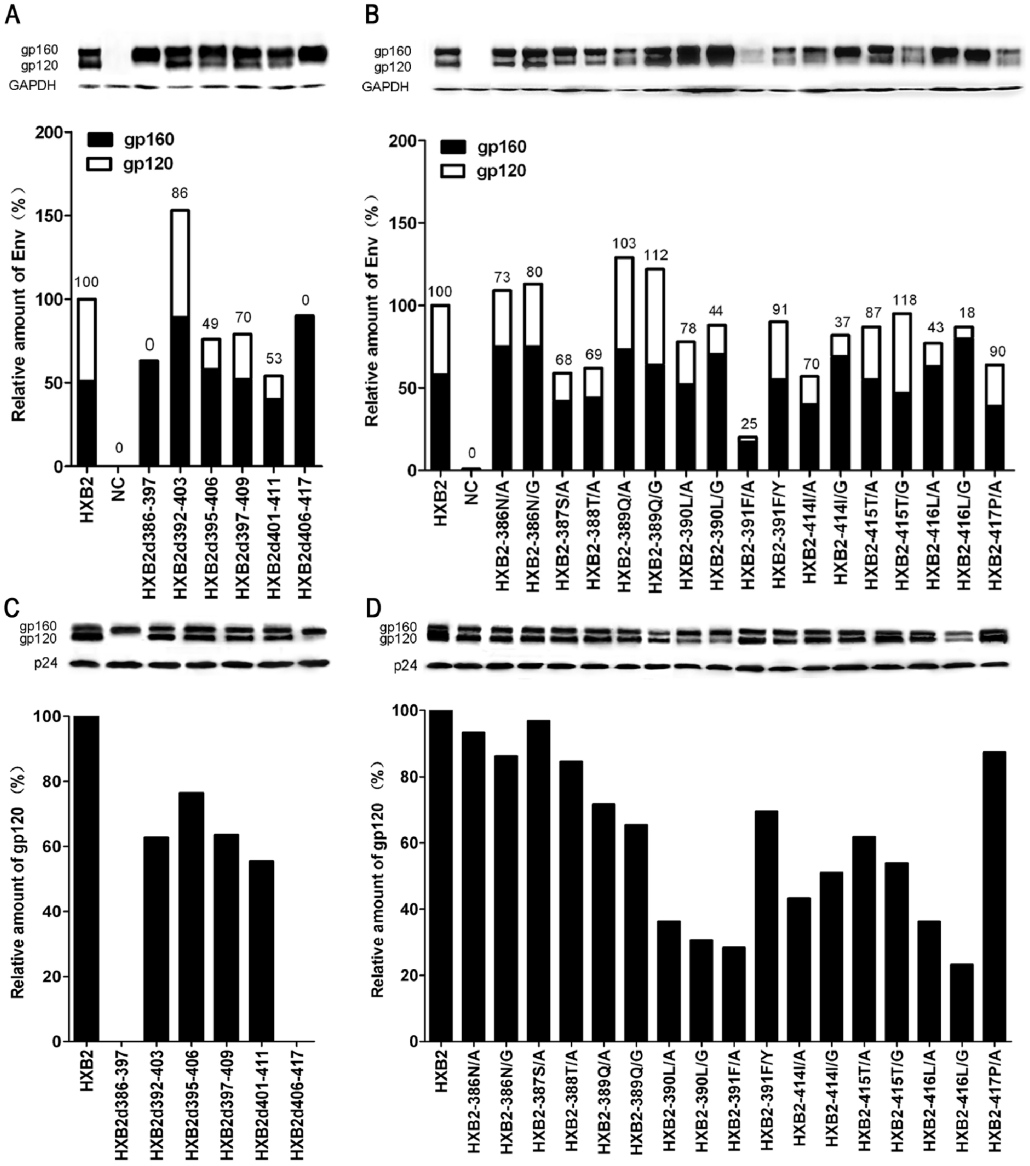
Cleavage efficiency and incorporation into a pseudovirus of the Env. Env expression in cell lysates (A for V4-deleted mutants and B for amino acid substitutions) and the pseudoviruses (C for V4-deleted mutants and D for amino acid substitutions) was determined. The supernatant of 293T cells was used as a negative control (no Env). The relative amount of total Env expression (gp160+ gp120) normalized to GAPDH in the cell lysates is shown as a percentage of HXB2-wt. The cleavage efficiency of gp160 is expressed as gp120/(gp120+ gp160) and shown on the top of the column (A, B). The gp120 incorporated into a pseudovirus was normalized to p24 and is shown as a percentage of HXB2-wt (C, D). One representative result of two to three independent transfections with each of the Env constructs is shown.

Because the deletion or substitution introduced into the V4 region caused a loss or decrease of viral infectivity, we were concerned whether the mutations affected the amount of Env on the pseudovirus surface as well as the expression and cleavage of the Env in the cells. Therefore, the amount of Env glycoprotein in the cell lysates (Figure 4A and 4B) and pseudoviruses (Figure 4C and 4D) was analyzed using western blot analysis. The effects of the mutations of V4 on Env expression, cleavage efficiency, and gp120 incorporated into the pseudoviruses are summarized in Table 1. We found that among all the mutants, only HXB2-391F/A exhibited a significantly lower expression of Env (20% of HXB2-wt) in cell lysates. The mutants HXB2d401-411, HXB2-387S/A, and HXB2-414I/A displayed 50–60% of wild-type Env expression. The remaining mutants did not present lower Env expression.

Because the presence of gp120 in cell lysates implies that gp160 is cleaved into gp120 and gp41, the amount of gp120 relative to the total amount of Env (gp120 plus gp160) in the cell lysates represents the cleavage efficiency. We found that the mutants that lost infectivity (HXB2d386-397, HXB2d406-417, and HXB2-391F/A) and those that had infectivity reduced to less than 20% of HXB2-wt (HXB2-390L/G, HXB2-414I/G and HXB2-416L/G) exhibited undetectable or greatly reduced cleavage levels of gp160 in the cell lysates (less than 50% of HXB2-wt).

Subsequently, western blot analysis of the presence of gp120 on purified viral particles was performed to probe for the effects on Env incorporation into virions. Since the gp120 detected in the cell culture supernatants represents mainly the shedding of gp120 (ultra-trace amounts of gp41 in culture supernatants, representing Env incorperated into virions, was detected, data not shown), we examined the gp120 expression in supernatants to investigate the shedding of Envs (normalized by P24). We found that the mutants with a reduced Env expression and/or cleavage of gp160 also displayed the reduced amount of virion-associated Env glycoproteins on the pseudoviruses.

HXB2d386-397 and HXB2d406-417 exhibited undetectable levels of gp120 in supernatants (Figure S1A) and incorporation into virions (Fig. 4C). HXB2-390L/G, HXB2-391F/A, and HXB2-416L/G exhibited lower levels of gp120 in supernatants and incorporation into the pseudovirions because there were greatly reduced levels of gp120 in supernatants and purified virions (less than 30% of HXB2-wt) (Figure S1B and Fig. 4D). It indicated that mutations with a reduced Env expression and/or cleavage of gp160 also reduced the amount of gp120 incorporation into the viral particles but not shedding.

We found that there was a positive correlation between the amount of gp120 on viral particles and infectivity among all the mutants with a single amino acid substitution (Correlation Coefficient, 0.561; P = 0.019) (Figure S2B). However, cleavage efficiency in cell lysates had no correlation with infectivity (Correlation Coefficient, 0.461; P = 0.061) (Figure S2A).

### Computational Analysis of the Mutants

**Figure 5:**
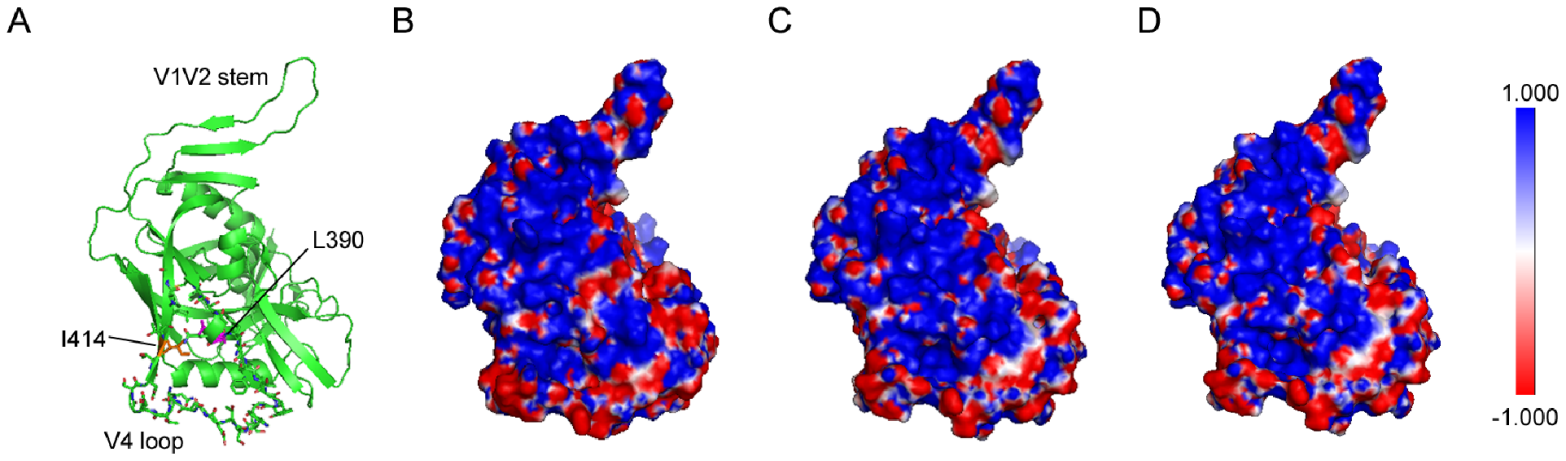
Distributions of the electrostatic potential on the gp120 molecule surface. The three-dimensional structure of HXB2 gp120 complexed with sCD4 and the CD4i-specific mAb 17b (PDB code:1GC1) is shown. The V4 loop is shown with sticks (A). The electrostatic surface potentials in the V4 loop side view of HXB2-wt (B), 390L/G (C), and 414I/G (D) are displayed. The negative (red) and the positive charges (blue) are shown.

**Figure 6:**
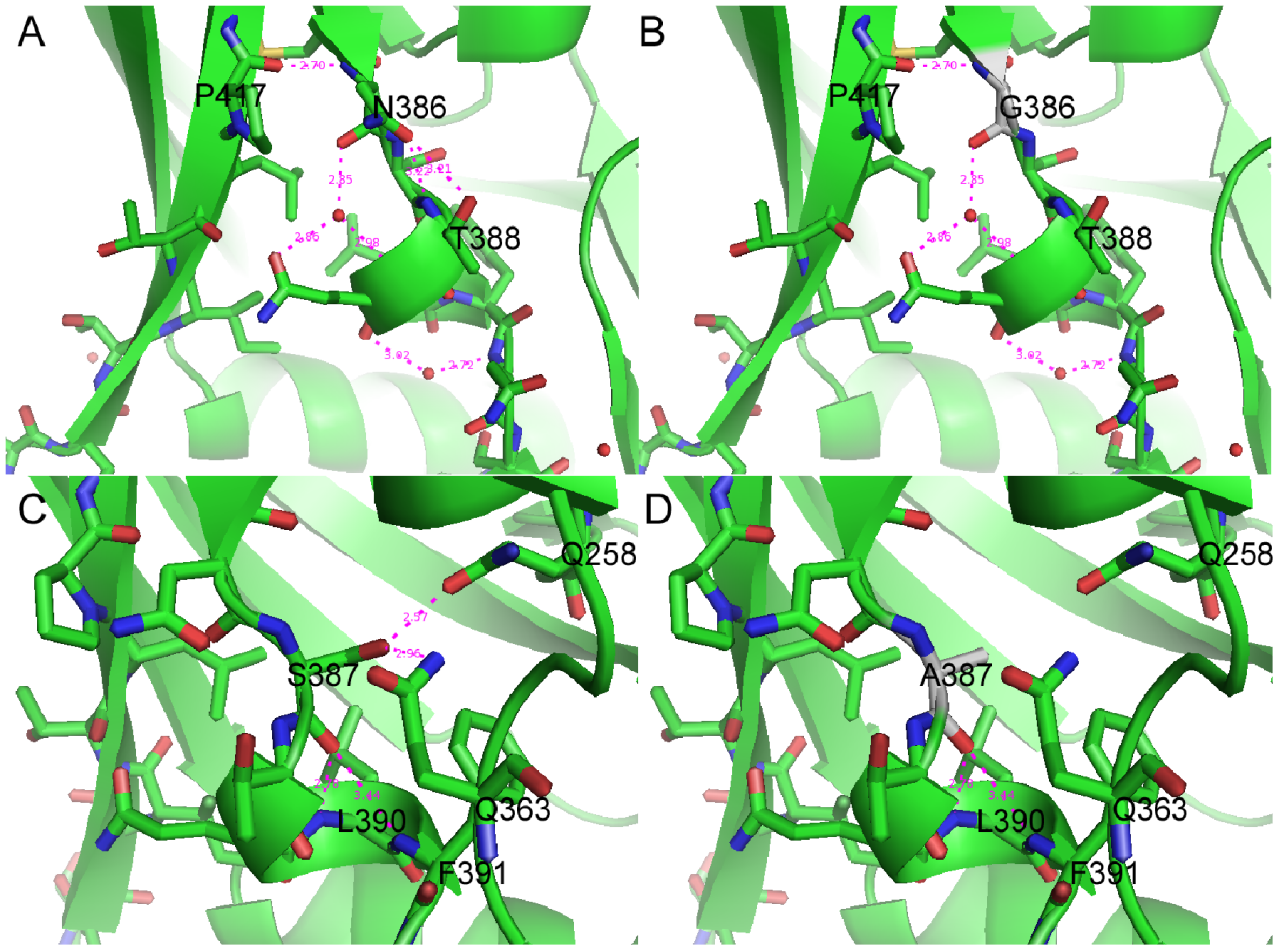
Model of residues 386 and 387 interaction with other residues. Amino acid residues in V4 of HXB2-wt or mutants and side-chain interactions are shown. A. 386N of HXB2-wt, B. mutant 386N/G, C. 387S of HXB2-wt, and D. mutant 387S/A. Potential hydrogen bonds are shown with magenta dashed lines.

To gain insights into how these mutations significantly affect viral infectivity or cleavage of Env, we modeled the structures of HXB2-wt and the mutants 390L/G, 391F/A, 414I/G, and 416L/G (the pseudovirus infectivity of which was significantly reduced, Figure 3), according to the crystal structure of an HIV gp120 Env glycoprotein in complex with sCD4/17b (PDB code 1GC1) [21] (Figures 5 and 6). We rebuilt the missing portion of V4 (aa397-409) at the energy-minimized structures of gp120 using the SWISS-MODEL server [22], [23]. The three-dimensional crystal structures of HXB2-wt were analyzed using PyMOL software and shown. We co-localized the models of each mutant (390L/G, 391F/A, 414I/G, and 416L/G) and HXB2-wt together and did not find significant conformational changes among them. This result suggested that these mutations did not or very slightly affected the overall structures.

In addition, the 390L/G, 391F/A, 414I/G, and 416L/G residues locate in two adjacent secondary structures of α4 and β19, respectively. Near these two secondary structures, a disulfide bond is formed between Cys385 and Cys418 residues located in β17 and β19 [21], respectively, which play an important role in stabilizing the conformations of both β17 and β19. Phenylalanine, leucine, and isoleucine residues are highly hydrophobic, and when the residues are replaced by alanine or glycine residue at positions 390, 391, 414, and 416, the hydrophobic properties of gp120 should be significantly changed and may induce the different distribution of the electrostatic potential of the gp120 surface. We found that the electrostatic surface potential of the HXB2-wt was remarkably different from those of the four mutants, 390L/G, 391F/A, 414I/G, and 416L/G, when viewed from the V4 loop side. The electrostatic surface potential of HXB2-wt and representative two mutants, 390L/G and 414I/G, are shown in Figure 5. The negative charge around the V4 loop in the HXB2-wt molecule surface was more exposed than those of the mutants (Figure 5B, 5C, and 5D).

Furthermore, we analyzed the models of each mutant that was fully characterized in terms of infectivity and protease cleavage (Figure 3E and Figure 4). HXB2-wt residue 386N interacts with 388T, 417P, and solvent water via strong hydrogen bond interactions. Although the substitution 386N/G removed the interaction with 388T and still kept other two interactions with C terminal of V4 loop and solvent water, the major structure did not change (Figure 6A and 6B). Therefore, the infectivity of mutant 386N/G was partially maintained (Figure 3). The mutant 417P/A interacts only with N386 via hydrogen bond interaction, and a substitution of 417P/A did not result in the removal of this interaction. Proline is a hydrophobic residue and contains the distinctive cyclic structure of side chain, giving proline an exceptional conformational rigidity. The substitution 417P/A might result in the conformational changing of the pocket of the V4 loop and affect virus entry. We found that 387S interacts not only with the residues L390 and F391 in the V4 loop but also with the 258Q in Loop B and 363Q located between β14 and β15 (Figure 6C and 6D). The 387S/A substitution removed the interaction with other parts of gp120 except the V4 loop. The mutant did not present a significant change of infectivity, suggesting the 387S/A substitution did not result in a conformational changing of α4. The mutation 389Q/G maintained the potential hydrogen bond with solvent water, and the glycine is smaller than glutamine, thus, steric hindrance might reduce the pocket formed by V4 (data not shown). The structures following the substitutions 390L/G, 391F/A, 414I/G and 416L/G did not appear to be significantly different compared with HXB2-wt, but the infectivity was dramatically reduced.

Using the same analysis methods, we investigated the trimer structures of HXB2 wild type and mutants (390L/G, 391F/A, 414I/G, and 416L/G) on the basis of the crystal structure (PDB code 3DNO) of the HIV-1 gp120 trimer in the CD4-bound state [24]. Because V4 region faces outside of trimer and each gp120 in the trimer separated from the others at more than 30 Å of shortest distance, the mutation within V4 did not affect the Env trimer structure (Figure S3).

## Discussion

During HIV-1 infection, because of the extensive virus evolution, coreceptor usage preference change from CCR5 to CXCR4 emerges in more than 50% of the infected individuals over time and is associated with progression to AIDS [25], [26]. We found in this work that V4 tended to have a shorter length, fewer PNGS, greater evolutionary distance, less negative net charge following the coreceptor usage preference change from CCR5 to CXCR4. These findings facilitate an understanding of the association of disease progression with the evolution of the V4 region. To date, the coreceptor binding interface of HIV-1 has only been partially identified. It is built up by the V3 stem-tip and some individual elements in C4 region [27]–[29]. The V4 region is a conformational neighbor to C4. In addition, previous findings have shown that rapid and extensive changes of V4-V5 can accompany coreceptor preference [15], [16] and that the N-linked glycan at position 413 in V4 may mask the epitopes near the base of V3 [30]. These findings suggest that there is possible direct or indirect disruption of the interface and a contribution of V4 to viral entry ability. We found that the mutant 417P/A, which has a mutation in a residue that is near to the coreceptor binding site [31], in the context ADA (a R5-tropic HIV-1 strain) obtained the ability to utilize CXCR4. We did not find other conserved amino acid residues in the V4 region that are responsible for the function.

Distinct regions of the Env are under selective pressures [3] and may tend to change to evade detection and elimination by the host immune system during the course of disease. Though the V4 region is highly polymorphic in length and amino acid sequence in HIV-1-infected individuals, the biological significance of its conserved N- and C-terminals has not been well studied. Our findings demonstrated that both the N- and C-terminals are important for maintaining HIV-1 infectivity in both CCR5- and CXCR4-tropic strains, and some highly conserved amino acids within these subregions are critical to maintain viral entry ability.

Notably, 390L, 391F (N-terminal), 414I, and 416L (C-terminal) are extremely conserved. The amino acid characteristics at these positions appear to be the main factor affecting viral infectivity because the mutations with different residues at one position demonstrated a different ability to maintain viral infectivity. For example, 390L/A maintained approximately 20% (ADA) and 60% (HXB2) of the viral infectivity of the wild type; however, 390L/G maintained less than 5%. In addition, 391F/Y maintained approximately 40% of the viral infectivity of the wild type, and 391F/A lost infectivity. The extreme sensitivity of these residues of gp120 to amino acid substitutions suggests that this region might be a potential target for development of new entry inhibitors that interrupt gp120 function and HIV entry via interacting with the conserved subregions.

In the V4 region, positions such as 390, 391, 414, and 416 in the N- or C-terminal may be critical for indirectly affecting the gp160 processing and the maturation of Env because the substitutions introduced into these position resulted in an significant decrease of cleavage of gp160 (<50%). In addition, the low cleavage efficiency and the subsequent insufficient incorporation of Env into viruses are responsible for the loss or decrease of the infectivity of the mutants. The events interrupting gp160 cleavage by the cellular furin-like proteases at the motif R-x-R/K-R [32] are poorly understood. It has been found that the different HIV-1 variants exhibit a range of cleavage efficiencies, even when all the variants possess identical cleavage sites [33]. Some evidences indicated that two regions at the N and C terminal of gpl20 associated with transmembrane glycoprotein gp4l. Positions within C1 (35W, 38V, 39Y, and 40Y) [34], C5 (491I, 494L, 496V, and 498P) [35] and mutants (256 S/Y, 262 N/T, 447 S/I, and 482/483/484 ELY/GRA) [36] have been found to be important for affecting gp160 processing. Changing residue W479 locating at the center of a hydrophobic which interface between layers 2 and 3 significantly disrupted the non-covalent association of gp120 with the Env trimer [37]. Moreover, a virus with an insertion of FLAG-tags into the N- or C-terminals of V4 loses infectivity completely and exhibits inefficient cleavage of the gp160 precursor [38]. However, introduction of a FLAG-tag into tip of V4 showed no deleterious effect with respect to protein expression and processing [39]. Our results revealed that the conserved positions at the N- and C-terminals of V4 (390L, 391F, 414I, and 416L) involved in gp160 processing and insufficient incorporation of Env into viruses, but not envelope shedding.

We further turned to high-resolution models of gp120 with its ligands to provide clues about how the viruses enter target cells while maintaining Env function. We focused on the mutations 390L/G, 391F/A, 414I/G, and 416L/G, which greatly reduced the cleavage efficiency of gp160. Among the mutants, the interactions between each mutation site and other residues in an objective are not changed compared with HXB2-wt either in the monomeric model or in the trimeric model. Therefore, the hydrophobicity of the amino acids leucine, isoleucine, and phenylalanine may play an important role in gp160 processing. These mutations result in a rearrangement of electrostatic potential on the gp120 molecule surface, including the surface around the V4 loop. The differences of charge distribution may significantly weaken the binding affinity of gp120 with other biologically functional molecules. In addition, these different distributions of the electrostatic surface potential of gp120 may affect the processing of the furin recognition site, although it is located in gp120 C5 region, which was not shown in the gp120 model. In addition, steric hindrance of the amino acids phenylalanine and proline may affect the conformational structure of V4 region and alter gp120 activity.

In summary, we have performed an extensive evolutionary analysis with Env variants containing partial deletions or mutations in the V4 region. The N- and C-terminals of the HIV-1 V4 region are highly conserved and critical to maintain virus entry ability. In addition, 390L, 391F, 414I, and 416L are critical to maintain gp160 processing. The hydrophobic properties and the electrostatic surface potential of gp120 with each mutant greatly contribute to this processing. The present work aids in the understanding of the functions of V4 in HIV-1 entry and also identifies a potential target for entry inhibitor development.

## Supporting Information

Figure S1 **Gp120 shedding in the supernatant.** The gp120 in the supernatants is normalized to p24 and is shown as a percentage of HXB2-wt (A, B). One representative result of two to three independent transfections with each of the Env constructs is shown.
(TIF)

Figure S2 **Correlation between infectivity of pseudoviruses and gp160 processing and incorporation.** The cleavage efficiency in cell lysates had no correlation with infectivity (Correlation Coefficient, 0.461; P = 0.061) (A), however, the infectivity positively is correlated with the amount of gp120 on viral particles (Correlation Coefficient, 0.561; P = 0.019) (B).
(TIF)

Figure S3 **Top views of the three-dimensional structure of HXB2 gp120 trimer in the CD4/17b-bound states (PDB code 3DNO).** We rebuilt the missing portion of V4 (aa397-409) at the energy-minimized structures of gp120 using the SWISS-MODEL server. The V4 loop is shown in magenta and V1/V2 stem regions are indicated in red.
(TIF)

Table S1 (DOC)
