# Supplementary figures and images for: Critical Amino Acids within the Human Immunodeficiency Virus Type 1 Envelope Glycoprotein V4 N- and C-Terminals Contribute to Virus Entry
Source: PLoS One. 2014 Jan 21;9(1):e86083. doi: 10.1371/journal.pone.0086083 (PMC3897638; doi:10.1371/journal.pone.0086083)

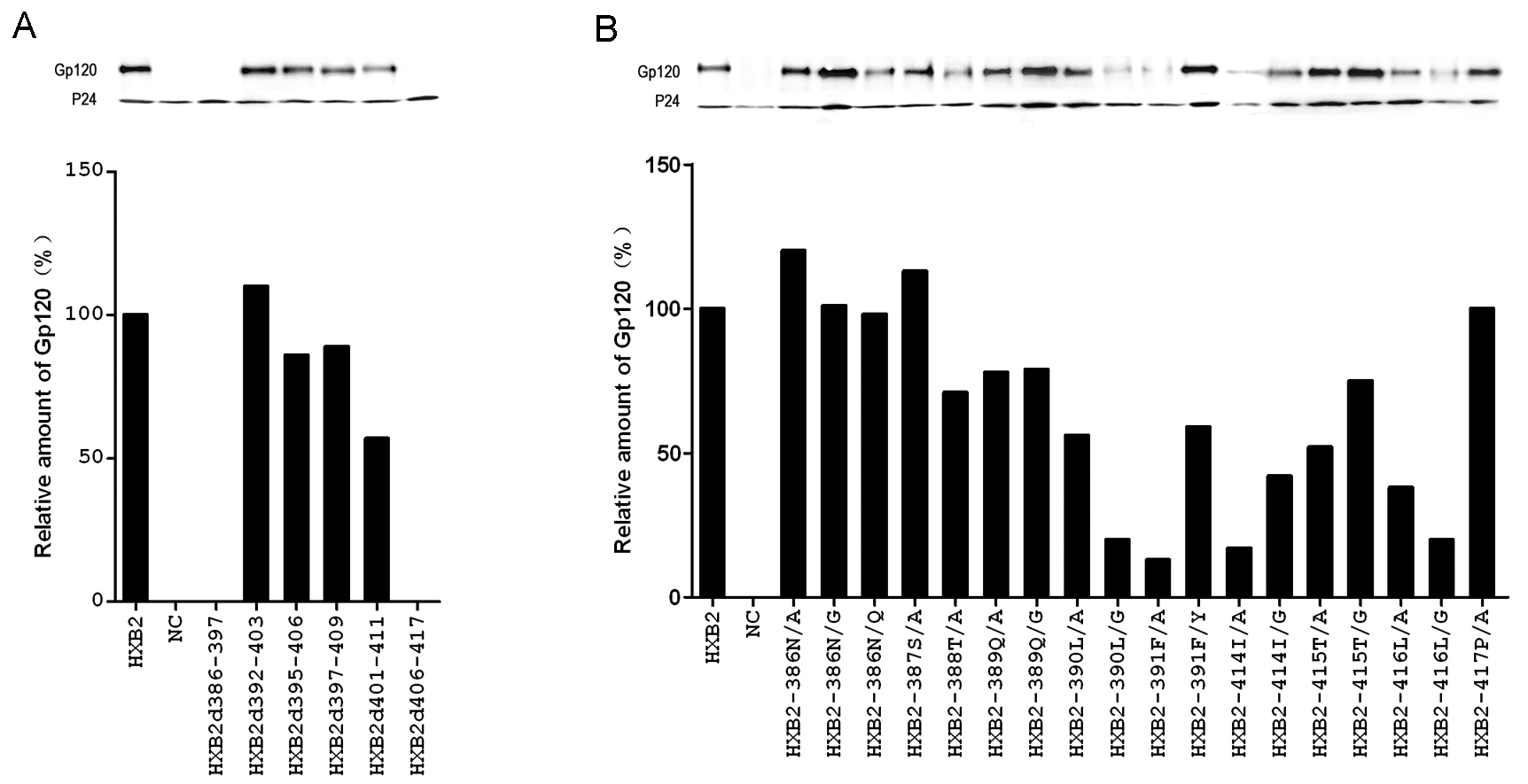

Supplement: Figure S1 — Gp120 shedding in the supernatant. The gp120 in the supernatants is normalized to p24 and is shown as a percentage of HXB2-wt (A, B). One representative result of two to three independent transfections with each of the Env constructs is shown. (TIF) [file pone.0086083.s001.tif]

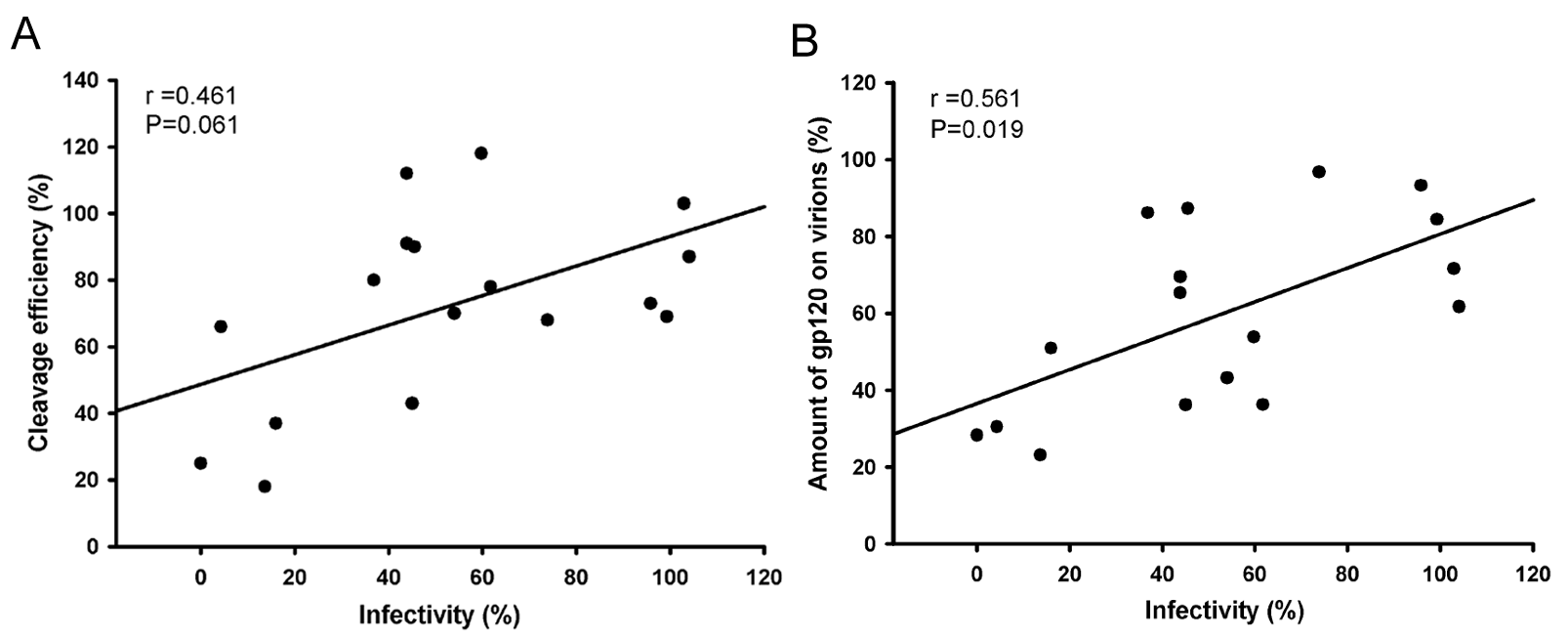

Supplement: Figure S2 — Correlation between infectivity of pseudoviruses and gp160 processing and incorporation. The cleavage efficiency in cell lysates had no correlation with infectivity (Correlation Coefficient, 0.461; P = 0.061) (A), however, the infectivity positively is correlated with the amount of gp120 on viral particles (Correlation Coefficient, 0.561; P = 0.019) (B). (TIF) [file pone.0086083.s002.tif]

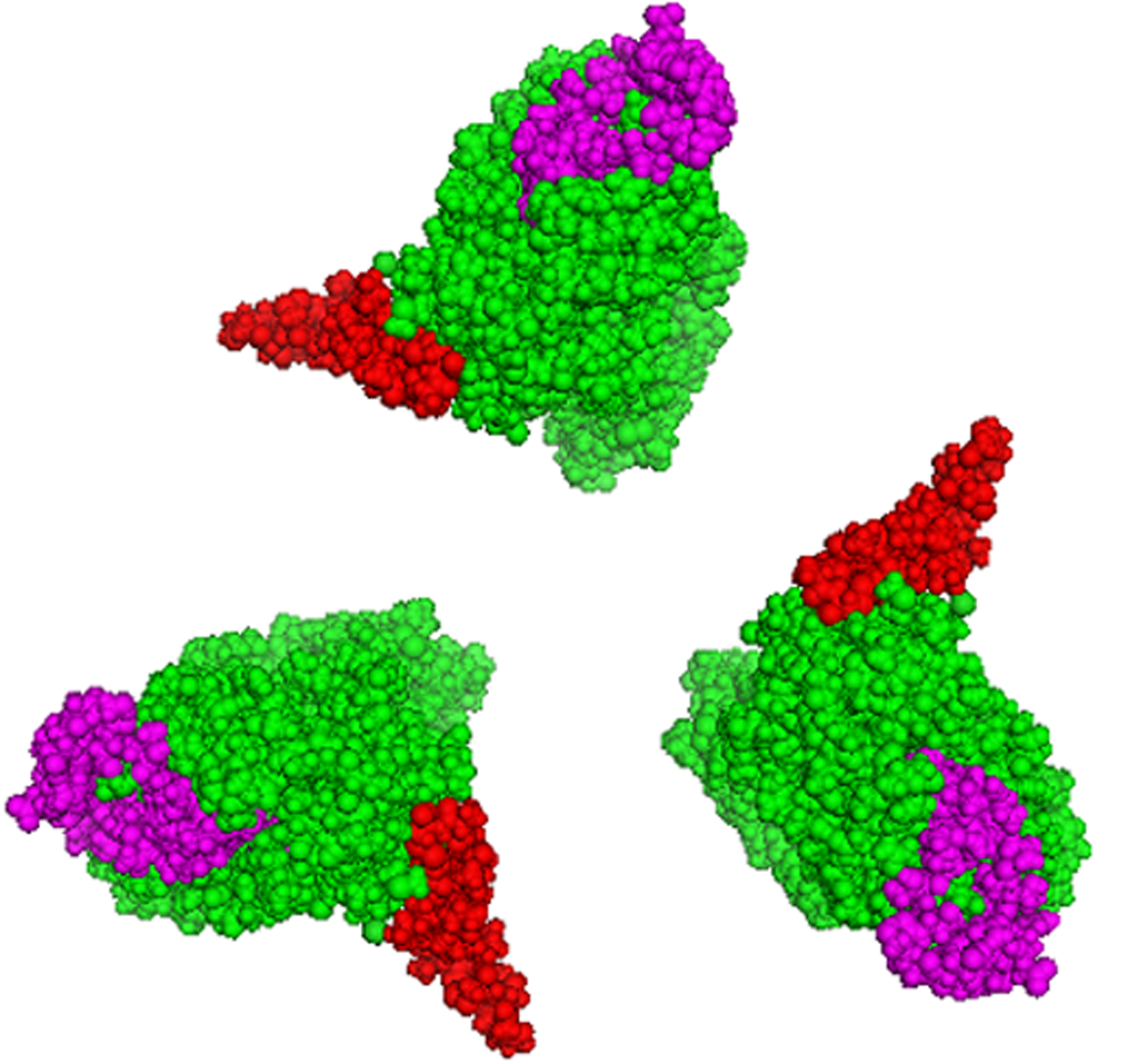

Supplement: Figure S3 — Top views of the three-dimensional structure of HXB2 gp120 trimer in the CD4/17b-bound states (PDB code 3DNO). We rebuilt the missing portion of V4 (aa397-409) at the energy-minimized structures of gp120 using the SWISS-MODEL server. The V4 loop is shown in magenta and V1/V2 stem regions are indicated in red. (TIF) [file pone.0086083.s003.tif]
