# Supplementary material for: Critical Amino Acids within the Human Immunodeficiency Virus Type 1 Envelope Glycoprotein V4 N- and C-Terminals Contribute to Virus Entry
Source: PLoS One. 2014 Jan 21;9(1):e86083. doi: 10.1371/journal.pone.0086083 (PMC3897638; doi:10.1371/journal.pone.0086083)
Supplement: Table S1 — (DOC) [file pone.0086083.s004.doc]

**Table S1.** Accession numbers of the V4 regions

| **Coreceptor usage** | **Accession number** | | | | |
| --- | --- | --- | --- | --- | --- |
| **CCR5-utilizing only**  **(n=190)** | AF112542 | AF112548 | AF112563 | AF112565 | AF254778 |
|  | AF254779 | AF254781 | AF310108 | AF310123 | AF491737 |
|  | AF491740 | AF541060 | AJ418478 | AJ418495 | AJ418514 |
|  | AJ418521 | AY426110 | AM156920 | AJ418531 | AJ810475 |
|  | AY669709 | AY173955 | AY173952 | AM156917 | AJ810480 |
|  | AY669711 | AY669713 | AY669725 | AY713408 | AY713410 |
|  | AY713411 | AY713412 | AY835434 | AY835436 | AY835437 |
|  | AY835438 | AY835439 | AY835440 | AY835441 | AY835442 |
|  | AY835443 | AY835446 | AY835447 | AY835448 | AY835449 |
|  | AY835450 | AY835451 | AY835452 | AY842786 | AY842808 |
|  | DQ177192 | DQ177191 | DQ177190 | DQ177188 | AY842824 |
|  | DQ177195 | DQ177198 | DQ177199 | DQ177200 | DQ177201 |
|  | DQ222211 | DQ178989 | DQ177210 | DQ177208 | DQ177207 |
|  | EU577135 | EU576936 | EU576991 | EU576909 | EU576584 |
|  | EF600083 | EF600091 | EF600099 | EF643652 | EF600075 |
|  | EF175209 | EF579969 | EF600067 | DQ869029 | DQ869026 |
|  | DQ869020 | DQ869033 | DQ869031 | DQ869030 | DQ869025 |
|  | DQ869021 | DQ869022 | DQ869024 | DQ869023 | DQ869019 |
|  | DQ869017 | DQ869015 | DQ869016 | DQ869014 | DQ645382 |
|  | EU576774 | EU576838 | EU576867 | EU576666 | EU576601 |
|  | EU576565 | EU576440 | EU576418 | EU576281 | EU576240 |
|  | EU576214 | EU575924 | EU575892 | EU575795 | EU575786 |
|  | EU575728 | EU575668 | EU575611 | EU575538 | EU575529 |
|  | EU575490 | EU575457 | EU575508 | EU575376 | EU575330 |
|  | EU575201 | EU575279 | EU575305 | EU575148 | EU575091 |
|  | EU272312 | EU575025 | EU575072 | EU272289 | EU272325 |
|  | EU272261 | EU272269 | EU272280 | EU272217 | EU272246 |
|  | EF643662 | EU272190 | EU272199 | EF643659 | EF643656 |
|  | U39258 | U66221 | U79719 | U79720 | U79721 |
|  | U32396 | U27443 | U23487 | U16217 | U08802 |
|  | U04908 | U08451 | U08801 | U08798 | U08450 |
|  | M38429 | M26727 | GU074012 | FJ798392 | FJ798329 |
|  | FJ653392 | FJ653356 | FJ653218 | FJ653194 | EU786680 |
|  | EU744146 | EU786676 | FJ653122 | FJ653098 | EU786678 |
|  | EU744055 | EU744097 | EU786677 | EU604591 | EU786679 |
|  | EU578657 | EU604550 | EU744014 | EU743973 | EU578340 |
|  | EU578419 | EU578539 | EU578561 | EU578380 | EU578309 |
|  | EU577288 | EU577329 | EU578272 | EU577210 | EU577190 |
| **CXCR4-utilizing only**  **(n=32)** | AF034383 | AF035534 | AY842819 | AY842799 | AY842825 |
|  | AF075720 | AF146728 | AF310132 | AJ810482 | A04321 |
|  | AY189526 | AY173956 | AY173951 | AM156919 | AM156915 |
|  | DQ286957 | DQ177202 | M19921 | U08447 | U48207 |
|  | EU604549 | EU578394 | DQ990880 | DQ869028 | DQ286958 |
|  | FJ653146 | FJ798323 | FJ798399 | FJ798416 | FJ798457 |
|  | FJ798527 | FJ798547 |  |  |  |
| **CCR5CXCR4-utilizing only (n=30)** | AJ810478 | AF310128 | AF310116 | AF286365 | AF035532 |
|  | AY842787 | AY842821 | AY842843 | DQ177203 | DQ177211 |
|  | EU578595 | EU272208 | EU272226 | DQ869032 | DQ869027 |
|  | EU786672 | EU272307 | EU272271 | EU272244 | EU272235 |
|  | M17451 | L22087 | K02007 | FJ798322 | FJ653254 |
|  | U08444 | U08445 | U08446 | U08796 | U66222 |
